# Co-detection of respiratory pathogens in children with *Mycoplasma pneumoniae* pneumonia: a multicenter study

**DOI:** 10.3389/fped.2025.1482880

**Published:** 2025-05-23

**Authors:** Xiaoyan Dong, Rui Li, Yingxue Zou, Lina Chen, Hailin Zhang, Fangfang Lyu, Wenhao Yang, Yanhua Niu, Haojie Wang, Run Guo, Xu Wang, Li Li, Zihao Lin, Li Luo, Danli Lu, Quan Lu, Hanmin Liu

**Affiliations:** ^1^ Department of Pulmonology, Shanghai Children’s Hospital, School of Medicine, Shanghai Jiao Tong University, Shanghai, China; ^2^ Department of Pulmonology, Tianjin Children’s Hospital (Children’s Hospital, Tianjin University), Machang Compus, Tianjin, China; ^3^ Department of Pediatric Pulmonology and Immunology, West China Second University Hospital, Sichuan University, Chengdu, China; ^4^ Department of Pediatric Respiratory Medicine, The Second Affiliated Hospital and Yuying Children’s Hospital of Wenzhou Medical University, Wenzhou, China; ^5^ Department of Pediatric Pulmonology, Yaan People’s Hospital, Yaan, China

**Keywords:** *Mycoplasma pneumoniae*, child, pneumonia, pathogen co-detection rate, targeted next-generation sequencing

## Abstract

**Objective:**

To investigate the prevalence and clinical significance of respiratory pathogen co-detection in children diagnosed with *Mycoplasma pneumoniae* pneumonia (MPP).

**Methods:**

A prospective observational multicenter study was conducted, collecting clinical data from pediatric patients diagnosed with MPP in four hospitals across China from December 1 to December 31, 2023. Targeted next-generation sequencing (tNGS) results and clinical characteristics were analyzed. Participants were divided into mild and severe groups according to disease severity. Severe cases were further subdivided into an MP alone group and a multi-pathogen co-detection group. Receiver operating characteristic (ROC) curve analysis was performed to assess the predictive performance of inflammatory biomarkers for multi-pathogen co-detection.

**Results:**

A total of 266 children were enrolled. Severe cases had significantly higher C-reactive protein (CRP), erythrocyte sedimentation rate (ESR), lactate dehydrogenase (LDH), D-dimer, interleukin-6 (IL-6), IL-10, and interferon-γ (IFN-γ) levels, as well as longer hospital stays (all *P* < 0.05). Multi-pathogen co-detection was found in 49.62% of MPP patients, and was more frequent in severe cases than in mild cases (54.05% vs. 39.51%, *P* < 0.05). The most common co-detected pathogens were rhinovirus, adenovirus, influenza A virus, *Haemophilus influenzae*, and *Streptococcus pneumoniae*. Among severe cases, the white blood cell (WBC) count and LDH, IL-6, and IL-10 levels were significantly higher in the multi-pathogen co-detection group compared to the MP alone group (*P* < 0.05).ROC analysis revealed that IL-6 and IL-10, especially in combination, effectively predicted multi-pathogen co-detection.

**Conclusions:**

Multi-pathogen co-infections substantially influence the severity of pediatric MPP. The findings highlight the diagnostic value of tNGS for identifying co-pathogens and underscore the predictive potential of inflammatory biomarkers (especially IL-6 and IL-10). The integration of tNGS and these biomarkers may facilitate early detection and targeted therapeutic interventions, thereby improving prevention and treatment outcomes in pediatric MPP.

## 1 Introduction

*Mycoplasma pneumoniae* (MP) is one of the principal pathogens responsible for community-acquired pneumonia (CAP) in children (1). MP spreads predominantly through respiratory droplets and exhibits cyclical outbreaks every 3–7 years, particularly in densely populated regions (2). While *Mycoplasma pneumoniae* pneumonia (MPP) is generally a self-limiting condition, severe cases can lead to significant complications, both within and outside the pulmonary system. These complications include necrotizing pneumonia, bronchiolitis obliterans, and acute respiratory distress syndrome, which can be life-threatening in severe cases (3, 4).

Co-infection with other pathogens in children with MPP is common, with reported co-infection rates ranging from 30% to 60% (5, 6). However, most previous studies were single-center or limited to certain pathogen types, providing insufficient comprehensive evidence regarding the clinical impact of co-infections on pediatric MPP severity.

In this prospective observational multicenter study, we utilized targeted next-generation sequencing (tNGS) to comprehensively detect respiratory co-pathogens among children hospitalized with MPP across different regions of China. By correlating pathogen profiles with clinical severity and inflammatory responses, we aimed to clarify how co-infections affect pediatric MPP outcomes. Additionally, receiver operating characteristic (ROC) curve analysis was conducted to determine whether inflammatory biomarkers could accurately predict multi-pathogen co-detection, thereby supporting early identification and optimized management strategies for Severe *Mycoplasma pneumoniae* pneumonia.

## 2 Method

### 2.1 Study patients

**Figure 1:**
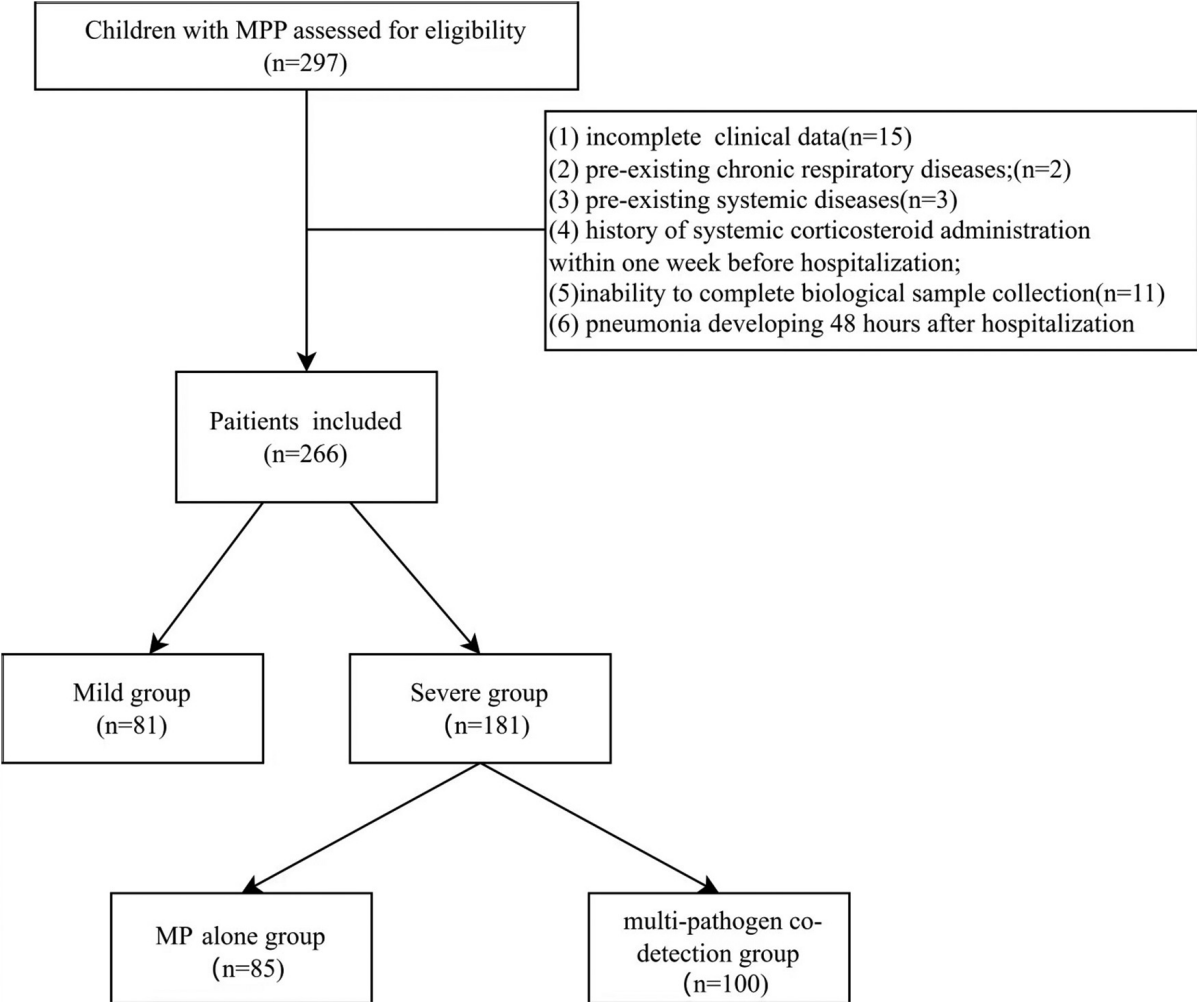
Flowchart of the study.

Clinical and laboratory data were prospectively collected from children with MPP who were hospitalized between December 1 and 31, 2023, at four centers in China: West China Second University Hospital (Chengdu), Tianjin Children's Hospital (Tianjin), Shanghai Children's Hospital (Shanghai), and Second Affiliated Hospital and Yuying Children’s Hospital of Wenzhou Medical University (Wenzhou). Initially, 297 patients were assessed for eligibility. After excluding 31 patients based on the exclusion criteria, a total of 266 patients were finally enrolled in the study (Figure 1).

Inclusion criteria: (1) presence of fever and respiratory symptoms; (2) pneumonia confirmed by physical examination and chest imaging; (3) laboratory-confirmed *Mycoplasma pneumoniae* infection.

Exclusion criteria: (1) incomplete clinical data; (2) pre-existing chronic respiratory diseases (e.g., bronchopulmonary dysplasia, primary ciliary dyskinesia, cystic fibrosis); (3) pre-existing systemic diseases (e.g., cardiovascular, hepatic, renal, hematologic, neurologic, or immunologic diseases); (4) history of systemic corticosteroid administration within one week before hospitalization; (5) inability to complete biological sample collection; (6) pneumonia developing 48 h after hospitalization.

### 2.2 Definitions

*Mycoplasma pneumoniae* pneumonia (MPP): Pneumonia accompanied by fever and respiratory symptoms, confirmed by physical examination and chest imaging, with laboratory-confirmed *Mycoplasma pneumoniae* infection.

Severe *Mycoplasma pneumoniae* pneumonia (SMPP) (7): Defined by the presence of one or more of the following criteria: (1) poor general condition; (2) altered consciousness; (3) cyanosis, dyspnea, pulse oxygen saturation ≤92%,or significant increase in breathing rate (>70 breaths/min in infants, >50 breaths/min in older children); (4) ultra-high fever or persistent high fever lasting more than 5 days; (5) refusal of food or signs of dehydration; (6) extensive pulmonary involvement (≥2/3 of a lobe or multilobar), pleural effusion, pneumothorax, atelectasis, pulmonary necrosis, or lung abscess; (7) extrapulmonary complications.

Based on disease severity, patients were classified into a mild group (uncomplicated MPP) and a severe group (meeting SMPP criteria). Severe cases were further subdivided according to pathogen detection results into an MP alone group (MP detected as the sole pathogen) and a multi-pathogen co-detection group (MP plus ≥1 additional respiratory pathogen detected).

### 2.3 Data collection

Clinical and microbiological data for hospitalized patients were obtained from the hospital clinical information system. Data included complete blood counts, CRP, ESR, liver function tests, other biochemical parameters, D-dimer, inflammatory cytokines, and length of hospital stay. For each patient, laboratory values at admission (within 24 h of hospitalization) were used as baseline values for comparison between groups.

### 2.4 Sample collection

Pharyngeal swabs and blood samples were collected within 24 h of hospital admission. In the mild group, trained physicians collected pharyngeal swabs by swabbing the back of the pharynx at least three times. In the severe group, bronchoalveolar lavage fluid (BALF) samples were collected during bronchoscopy within 7 days after admission. The pharyngeal swabs and BALF samples were stored at −80°C and transported to a designated laboratory for targeted next-generation sequencing (tNGS) analysis at ≤−20°C.

### 2.5 Targeted next-generation sequencing

Pharyngeal swabs (mild group) and BALF samples (severe group) were transported to a central laboratory for targeted next-generation sequencing (tNGS) analysis (Respiratory 100 panel, KingMed Diagnostics, Guangzhou, China). According to the manufacturer's instructions, the tNGS method enables the early diagnosis of respiratory infections by detecting 198 pathogens. After nucleic acid extraction, specific primers targeting bacterial, mycobacterial, viral, fungal, and selected antimicrobial resistance sequences were used to enrich multiplex targets, followed by library preparation for NGS sequencing (8, 9). The reads of sequence (normalized sequence number in 100,000 primary sequences detected) shown in the report were calculated based on internal control. When estimating the concentration of the target pathogen, exogenous plasmids of known concentrations were used as the internal control. The number of normalized sequence reads of the target pathogen could be calculated using the amplification efficiency ratio of the internal control.

To ensure accurate clinical interpretation, all tNGS results were independently reviewed by two experienced clinicians, who carefully assessed each detected pathogen in the context of the patient’s medical history, symptoms, imaging findings, and laboratory results to distinguish true infections from colonization. Given that pharyngeal swabs may detect upper airway commensals, independent interpretation by two clinicians helped ensure accurate pathogen identification. BALF samples from severe cases, representing direct lower respiratory specimens, further reduced concerns about colonization and enhanced diagnostic accuracy.

### 2.6 Statistical analysis

Data analyses were performed using SPSS software (version 22.0). Normally distributed continuous data were presented as mean ± standard deviation (SD) and compared using independent-sample *t*-tests. Skewed distribution data were reported as medians with interquartile ranges (IQR) and analyzed using Mann–Whitney *U* tests. Categorical variables were presented as frequencies and percentages, with comparisons between groups performed using the chi-square test or Fisher's exact test. ROC curve analysis was performed to evaluate the predictive value of inflammatory biomarkers for identifying multi-pathogen co-detection. A Sankey diagram was constructed to visualize the distribution of respiratory pathogens co-detected with MP. The difference was considered statistically significant at *P* < 0.05.

## 3 Results

### 3.1 Demographic and clinical characteristics of children with MPP

A total of 266 children with MPP were enrolled in this study (119 males and 147 females), including 185 severe cases and 81 mild cases. The median age at onset was 7.0 years in both groups, with no significant differences in age or gender distribution between severe and mild cases (*P* > 0.05 for both). However, patients in the severe group had significantly longer hospital stays (*P* < 0.001; Table 1).

**Table 1:** Comparison of clinical characteristics between severe and mild groups.

Characteristic	Severe group (*n* = 185)	Mild group (*n* = 81)	*P*-value
Demographic characteristics			
Male	82 (44.3)	37 (45.7)	0.894
Age (years)	7.0 (6.0, 9.0)	7.0 (4.5, 9.0)	0.705
Laboratory findings			
WBC (×10^9^/L)	7.01 (5.80, 8.80)	7.28 (5.57, 9.01)	0.795
Neutrophil ratio (%)	64.05 (53.82, 72.67)	59.15 (49.95, 72.52)	0.149
Hemoglobin (g/L)	125.31 ± 9.73	128.56 ± 11.19	0.022
CRP (mg/L)	15.0 (6.65, 31.00)	6.15 (5.00, 14.75)	<0.001
D-dimer (mg/L)	0.64 (0.36, 1.09)	0.48 (0.31, 0.73)	0.008
ESR (mm/h)	44.0 (33.0, 61.75)	35.0 (24.75, 49.25)	0.042
ALT (U/L)	15.0 (12.0, 22.0)	14.0 (11.0, 17.0)	0.224
LDH (U/L)	328.0 (277.0, 396.0)	299.0 (253.0, 334.5)	0.003
IL-1β (pg/ml)	11.39 (2.70, 43.90)	11.67 (3.21, 37.80)	0.925
IL-2 (pg/ml)	3.37 (2.44, 16.00)	2.50 (2.44, 18.60)	0.130
IL-4 (pg/ml)	3.51 (2.50, 38.85)	3.15 (2.50, 37.30)	0.325
IL-5 (pg/ml)	9.62 (2.68, 248.47)	4.69 (2.77, 307.0)	0.617
IL-6 (pg/ml)	58.64 (27.02, 130.91)	27.75 (4.10, 126.45)	0.003
IL-8 (pg/ml)	36.86 (13.09, 62.65)	26.22 (12.99, 56.52)	0.291
IL-10 (pg/ml)	21.00 (9.07, 45.50)	8.46 (2.44, 32.60)	<0.001
IL-12p70 (pg/ml)	2.50 (2.44, 4.80)	2.90 (2.44, 4.00)	0.412
IL-17 (pg/ml)	7.39 (1.23, 49.47)	8.42 (2.47, 51.30)	0.878
IFN-γ (pg/ml)	40.00 (12.23, 255.15)	17.52 (10.00, 157.27)	0.043
TNF-α (pg/ml)	5.18 (2.50, 85.30)	4.97 (2.58, 69.00)	0.644
Clinical course			
Onset-to-admission time (days)	7.0 (5.0, 10.0)	7.0 (6.0, 9.0)	0.935
Hospital stay (days)	7.0 (5.0, 8.0)	5.0 (4.0, 7.0)	<0.001

Data are presented as median (IQR) for continuous variables, except hemoglobin (mean ± SD). *P* values were calculated by chi-square test for categorical variables (sex), independent *t*-test for hemoglobin, and Mann–Whitney *U* tests for other continuous variables. *P* values <0.05 indicate differences between severe and mild MPP groups.

MPP, *Mycoplasma pneumoniae* pneumonia; WBC, white blood cell count; CRP, C-reactive protein; ESR, erythrocyte sedimentation rate; ALT, alanine aminotransferase; LDH, lactate dehydrogenase; IL, interleukin; IFN-γ, interferon-gamma; TNF-α, tumor necrosis factor-alpha.

Compared to the mild group, patients in the severe group exhibited significantly higher levels of inflammatory biomarkers (CRP, ESR, LDH, D-dimer) and serum cytokines (IL-6, IL-10, IFN-γ) (all *P* < 0.05; Table 1).

### 3.2 Detection and regional distribution of respiratory pathogens in children with MPP

**Figure 2:**
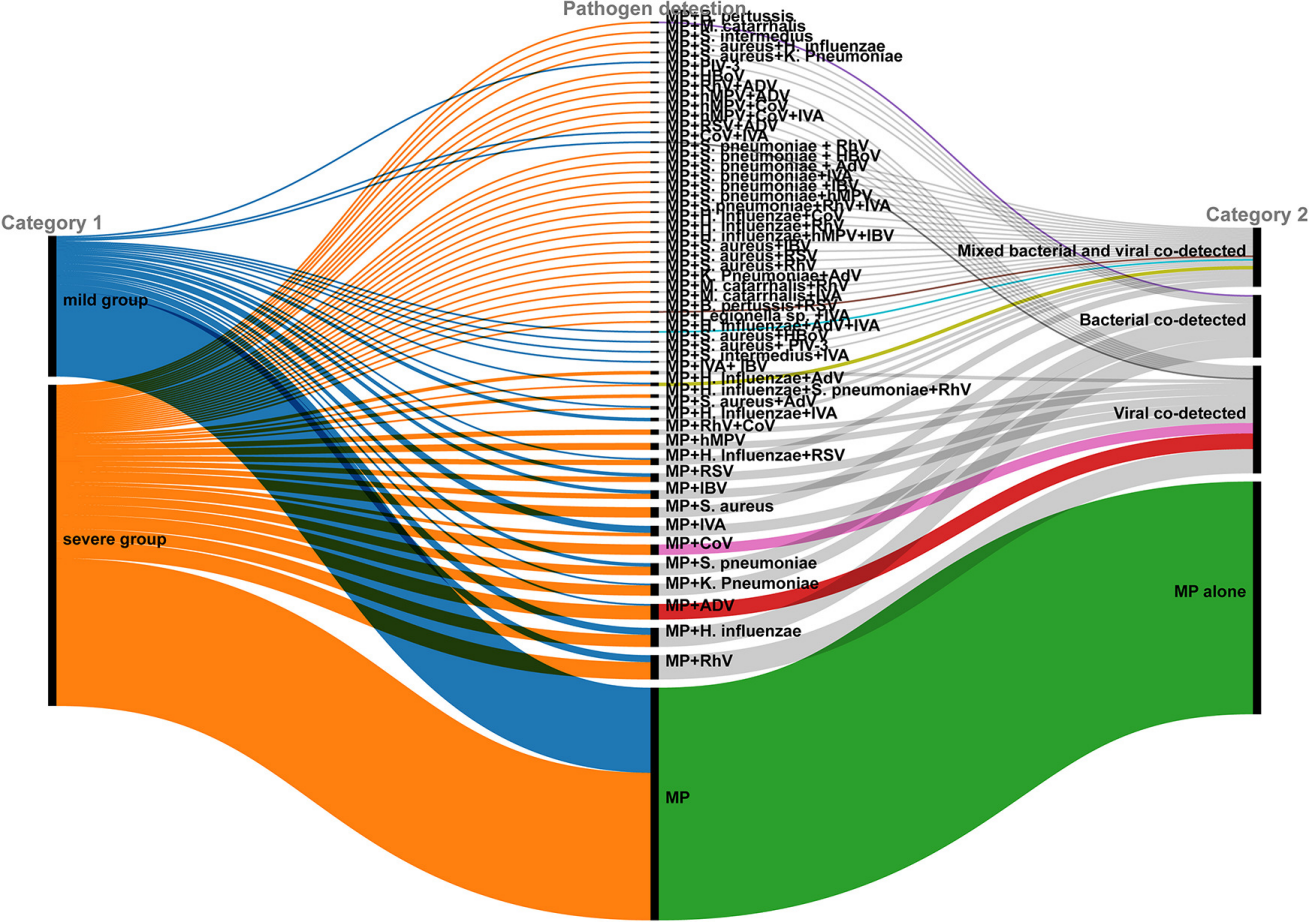
Respiratory pathogens co-detected with MP in children: severe vs. mild groups.

Respiratory pathogens other than MP were identified in 132 out of 266 children (49.62%). Multi-pathogen co-detection rate was significantly higher in the severe group compared to the mild group (54.05% vs. 39.51%, *χ*^2^ = 4.769, *P* = 0.033), especially bacterial co-detection rate (*P* < 0.001; Table 2). A Sankey diagram further illustrated clear differences in respiratory pathogen profiles between severe and mild groups (Figure 2).

**Table 2:** Respiratory pathogens co-detected with MP: comparison between severe and mild groups.

Pathogen detection	Severe group (*n* = 185)	Mild group (*n* = 81)	*P*-value
MP alone	85 (45.95)	49 (60.49)	0.033
MP co-detection (≥1 additional pathogen)			
Bacterial co-detected	28 (15.14)	8 (9.88)	<0.001
*S. pneumoniae*	5 (2.70)	2 (2.47)	—
*H. influenzae*	7 (3.78)	4 (4.94)	—
*S. aureus*	6 (3.24)	0 (0.00)	—
*K. pneumoniae*	6 (3.24)	1 (1.23)	—
*B. pertussis*	1 (0.54)	0 (0.00)	—
*M. catarrhalis*	1 (0.54)	0 (0.00)	—
*S. intermedius*	1 (0.54)	0 (0.00)	—
*S. aureus* *+* *H. influenzae*	1 (0.54)	0 (0.00)	—
*S. aureus* *+* *K. pneumoniae*	0 (0.00)	1 (1.23)	—
Viral co-detected	47 (25.41)	15 (18.52)	0.271
RhV	10 (5.41)	4 (4.94)	—
RSV	3 (1.62)	2 (2.47)	—
AdV	8 (4.32)	1 (1.23)	—
IAV	2 (1.08)	4 (4.94)	—
IBV	3 (1.62)	2 (2.47)	—
PIV-3	1 (0.54)	0 (0.00)	—
CoV	6 (3.24)	0 (0.00)	—
hMPV	4 (2.16)	0 (0.00)	—
HBoV	1 (0.54)	0 (0.00)	—
RhV + CoV	3 (1.62)	0 (0.00)	—
RhV + AdV	1 (0.54)	0 (0.00)	—
hMPV + AdV	1 (0.54)	0 (0.00)	—
RSV + AdV	0 (0.00)	1 (1.23)	—
hMPV + CoV	1 (0.54)	0 (0.00)	—
hMPV + CoV + IAV	1 (0.54)	0 (0.00)	—
IAV + IBV	2 (1.08)	0 (0.00)	—
CoV + IAV	0 (0.00)	1 (1.23)	—
Mixed bacterial and viral co-detected	25 (13.51)	9 (11.11)	0.562
*S. pneumoniae* + RhV	1 (0.54)	0 (0.00)	—
*S. pneumoniae* + HBoV	1 (0.54)	0 (0.00)	—
*S. pneumoniae* + AdV	1 (0.54)	0 (0.00)	—
*S. pneumoniae* + IAV	1 (0.54)	0 (0.00)	—
*S. pneumoniae* + IBV	1 (0.54)	0 (0.00)	—
*S. pneumoniae* + hMPV	1 (0.54)	0 (0.00)	—
*S. pneumoniae* + RhV + IAV	1 (0.54)	0 (0.00)	—
*H. influenzae* + RSV	3 (1.62)	1 (1.23)	—
*H. influenzae* + AdV	1 (0.54)	1 (1.23)	—
*H. influenzae* + AdV + IAV	0 (0.00)	1 (1.23)	—
*H. influenzae* + CoV	1 (0.54)	0 (0.00)	—
*H. influenzae* + RhV	1 (0.54)	0 (0.00)	—
*H. influenzae* + IAV	0 (0.00)	2 (2.47)	—
*H. influenzae* + *S. pneumoniae* + RhV	2 (1.08)	0 (0.00)	—
*H. influenzae* + hMPV + IBV	1 (0.54)	0 (0.00)	—
*S. aureus* + IBV	1 (0.54)	0 (0.00)	—
*S. aureus* + AdV	1 (0.54)	1 (1.23)	—
*S. aureus* + RSV	1 (0.54)	0 (0.00)	—
*S. aureus* + RhV	1 (0.54)	0 (0.00)	—
*S. aureus* + HBoV	0 (0.00)	1 (1.23)	—
*S. aureus* + PIV-3	0 (0.00)	1 (1.23)	—
*K. pneumoniae* + AdV	1 (0.54)	0 (0.00)	—
*M. catarrhali*s + RhV	1 (0.54)	0 (0.00)	—
*M. catarrhalis* + IAV	1 (0.54)	0 (0.00)	—
*B. pertussis* + RSV	1 (0.54)	0 (0.00)	—
*Legionella sp*. + IAV	0 (0.00)	1 (1.23)	—

Values are number of cases (percentage of group). *P* values < 0.05 indicate a significant difference between severe and mild groups. No statistical tests were conducted for individual pathogens due to small subgroup sizes. MP co-detection refers to detection of ≥1 additional respiratory pathogen along with *Mycoplasma pneumoniae*.

MP, *Mycoplasma pneumoniae*; *S. pneumoniae*, *Streptococcus pneumoniae*; *H. influenzae*, *Haemophilus influenzae*; *S. aureus*, *Staphylococcus aureus*; *K. pneumoniae*, *Klebsiella pneumoniae*; *B. pertussis*, *Bordetella pertussis*; *M. catarrhalis*, *Moraxella catarrhalis*; *S. intermedius*, *Streptococcus intermedius*; *L. pneumophila*, *Legionella pneumophila*; RhV, rhinovirus; RSV, respiratory syncytial virus; AdV, adenovirus; HBoV, human bocavirus; PIV-3, parainfluenza virus type 3; CoV, coronavirus; hMPV, human metapneumovirus; IAV, influenza A virus; IBV, influenza B virus.

The tNGS analysis identified multi-pathogen co-detection in 132 children (49.62%). The most commonly co-detected organisms were *Haemophilus influenzae* (*H. influenzae*, 26 cases, 9.8%), rhinovirus (RhV, 25 cases, 9.4%), adenovirus (AdV, 19 cases, 7.1%), influenza A virus (IAV, 18 cases, 6.8%), *Streptococcus pneumoniae* (*S. pneumoniae*, 16 cases, 6.0%), *Staphylococcus aureus* (*S. aureus*, 15 cases, 5.6%), and human coronavirus (CoV, 13 cases, 4.9%). RhV, AdV, and IAV were the predominant viral pathogens, while *H. influenzae* and *S. pneumoniae* were the primary bacterial pathogens co-detected with MP. Although detection rates of AdV, CoV, RhV, human metapneumovirus (hMPV), influenza B virus (IBV), *S. pneumoniae* and *S. aureus* were higher in the severe group than in the mild group, these differences were not statistically significant (Table 3).

**Table 3:** Detection rates of specific pathogens co-detected with MP: comparison between severe and mild groups.

Pathogen	Severe group (*n* = 185)	Mild group (*n* = 81)	*P*-value
AdV	14 (7.68)	5 (6.17)	0.800
CoV	12 (6.49)	1 (1.23)	0.074
RhV	21 (11.35)	4 (4.94)	0.114
*S. pneumoniae*	14 (7.57)	2 (2.47)	0.184
IAV	9 (4.87)	9 (11.11)	0.069
hMPV	9 (4.87)	0 (0)	0.099
RSV	8 (4.32)	4 (4.94)	1.000
IBV	8 (4.32)	2 (2.47)	0.703
*S. aureus*	11 (5.94)	4 (4.94)	0.969
*H. influenzae*	17 (9.19)	9 (11.11)	0.656

Values are number of cases with pathogen detected (percentage of group). *P* values were calculated by chi-square test or Fisher’s exact test. AdV, adenovirus; CoV, coronavirus; RhV, rhinovirus; *S. pneumoniae*, *Streptococcus pneumoniae*; IAV, influenza A virus; hMPV, human metapneumovirus; RSV, respiratory syncytial virus; IBV, influenza B virus; *S. aureus*, *Staphylococcus aureus*; *H. influenzae*, *Haemophilus influenzae*.

No significant differences were observed in the rates of multi-pathogen co-detection with MP between mild and severe cases across different regions (all *P* > 0.05; Table 4). However, the predominant pathogens detected varied by region, with *H. influenzae* being most common in Tianjin (13.33%), AdV and IAV in Shanghai (each 12.65%), and RhV predominating in both Wenzhou (18.42%) and Chengdu (12.36%). The detection rates of RhV and *S. aureus* differed significantly among the four regions (*P* < 0.05; Table 5).

**Table 4:** Comparison of multi-pathogen co-detection rates between mild and severe cases of MPP across different regions.

Region	Mild cases MP co-detection, *n* (%)	Severe cases MP co-detection, *n* (%)	*P*-value
Tianjin	7/20 (35.0)	16/40 (40.0)	0.783
Shanghai	14/24 (58.3)	33/55 (60.0)	1.000
Wenzhou	3/8 (37.5)	21/30 (70.0)	0.117
Chengdu	8/29 (27.6)	30/60 (50.0)	0.067

Data presented as number/total number (%). MP co-detection refers to detection of ≥1 additional respiratory pathogen along with *Mycoplasma pneumoniae*. *P* values were calculated by chi-square test or Fisher’s exact test. MP, *Mycoplasma pneumoniae*.

**Table 5:** Detection rates of specific pathogens co-detected with MP across different regions.

Pathogen	Tianjin (*n* = 60)	Shanghai (*n* = 79)	Wenzhou (*n* = 38)	Chengdu (*n* = 89)	*P*-value
AdV	5 (8.33)	10 (12.66)	2 (5.26)	2 (2.25)	0.056
CoV	0 (0)	4 (5.06)	1 (2.63)	8 (8.99)	0.066
RhV	1 (1.67)	6 (7.59)	7 (18.42)	11 (12.34)	0.027
*S. pneumoniae*	3 (5.00)	6 (7.59)	3 (7.89)	4 (4.49)	0.777
IAV	1 (1.67)	10 (12.66)	2 (5.26)	5 (5.62)	0.079
hMPV	1 (1.67)	3 (3.80)	3 (7.89)	2 (2.25)	0.367
RSV	4 (6.67)	1 (1.27)	0 (0)	7 (7.87)	0.074
IBV	3 (5.00)	2 (2.53)	1 (2.63)	4 (4.49)	0.885
*S. aureus*	0 (0)	7 (8.86)	6 (15.79)	2 (2.25)	0.002
*H. influenzae*	8 (13.33)	7 (8.86)	2 (5.26)	9 (10.11)	0.610

Values are number of cases with pathogen detected (% of cases in that region). *P* values were calculated by chi-square test or Fisher’s exact test. AdV, adenovirus; CoV, coronavirus; RhV, rhinovirus; *S. pneumoniae*, *Streptococcus pneumoniae*; IAV, influenza A virus; hMPV, human metapneumovirus; RSV, respiratory syncytial virus; IBV, influenza B virus; *S. aureus*, *Staphylococcus aureus*; *H. influenzae*, *Haemophilus influenzae*.

### 3.3 Demographic and clinical characteristics of SMPP patients in the MP alone and multi-pathogen co-detection groups

Among the 185 children with SMPP, 85 had MP detected alone and 100 had co-detection of additional pathogens. No significant differences were observed between the two groups regarding gender distribution, age of onset, time from symptom onset to admission, or length of hospital stay (all *P* > 0.05; Table 6).

**Table 6:** Clinical characteristics of children with SMPP: comparison between the MP alone and multi-pathogen co-detection groups.

Characteristic	MP alone group (*n* = 85)	Multi-pathogen Co-detection group (*n* = 100)	*P*-value
Demographic characteristics			
Male, *n* (%)	39 (45.9)	43 (43.0)	0.767
Age (years),	7.0 (6.0, 9.0)	7.0 (6.0, 8.0)	0.091
Laboratory findings			
WBC (×10^9^/L)	6.80 (5.63, 7.90)	7.37 (5.90, 9.58)	0.023
Neutrophil ratio (%)	63.90 (53.5, 72.25)	64.20 (53.90, 73.30)	0.799
Hemoglobin (g/L)	126.24 ± 9.34	124.53 ± 10.08	0.237
CRP (mg/L)	15.00 (7.00, 36.60)	14.50 (5.97, 28.09)	0.444
D-dimer (mg/L)	0.63 (0.42, 1.08)	0.68 (0.41, 1.39)	0.638
ESR (mm/h)	44.50 (34.00, 60.50)	44.00 (33.00, 64.00)	0.789
ALT (U/L)	14.00 (11.00, 18.50)	16.00 (13.00, 24.50)	0.053
LDH (U/L)	312.00 (266.00, 360.50)	344.00 (282.00, 414.50)	0.041
IL-1β (pg/ml)	10.27 (2.96, 51.49)	14.49 (3.84, 44.32)	0.609
IL-2 (pg/ml)	3.46 (2.50, 11.25)	3.10 (2.44, 15.50)	0.642
IL-4 (pg/ml)	3.42 (2.50, 40.00)	3.65 (2.50, 33.97)	0.758
IL-5 (pg/ml)	41.30 (3.25, 300.50)	21.29 (5.89, 350.60)	0.251
IL-6 (pg/ml)	30.02 (14.12, 103.70)	69.00 (45.71, 114.25)	<0.001
IL-8 (pg/ml)	26.33 (16.68, 68.24)	26.90 (16.26, 59.63)	0.673
IL-10 (pg/ml)	11.40 (5.58, 29.42)	22.46 (18.00, 45.12)	<0.001
IL-12p70 (pg/ml)	2.50 (2.44, 3.49)	2.56 (2.44, 10.55)	0.369
IL-17 (pg/ml)	5.15 (1.09, 59.40)	9.61 (2.92, 35.30)	0.644
IFN-γ (pg/ml)	45.07 (12.23, 266.32)	51.70 (19.01, 462.40)	0.077
TNF-α (pg/ml)	6.63 (2.55, 88.50)	7.60 (2.50, 89.70)	0.957
Clinical course			
Onset-to-admission time (days)	6.0 (5.0, 8.0)	7.0 (6.0, 10.0)	0.069
Hospital stay (days)	7.0 (5.0, 8.0)	7.0 (5.0, 8.5)	0.580

Data are presented as median (IQR) for continuous variables, except hemoglobin (mean ± SD). *P* values were calculated by chi-square test for categorical variables (sex), independent *t*-test for hemoglobin, and Mann–Whitney *U* tests for other continuous variables. MP, *Mycoplasma pneumoniae*; WBC, white blood cell count; CRP, C-reactive protein; ESR, erythrocyte sedimentation rate; ALT, alanine aminotransferase; LDH, lactate dehydrogenase; IL, interleukin; IFN-γ, interferon-gamma; TNF-α, tumor necrosis factor-alpha.

However, patients in the multi-pathogen co-detection group had significantly higher WBC counts and higher LDH, IL-6, and IL-10 levels compared to those in the MP alone group (all *P* < 0.05; Table 6).

### 3.4 ROC analysis of inflammatory markers for predicting co-detection in SMPP

**Figure 3:**
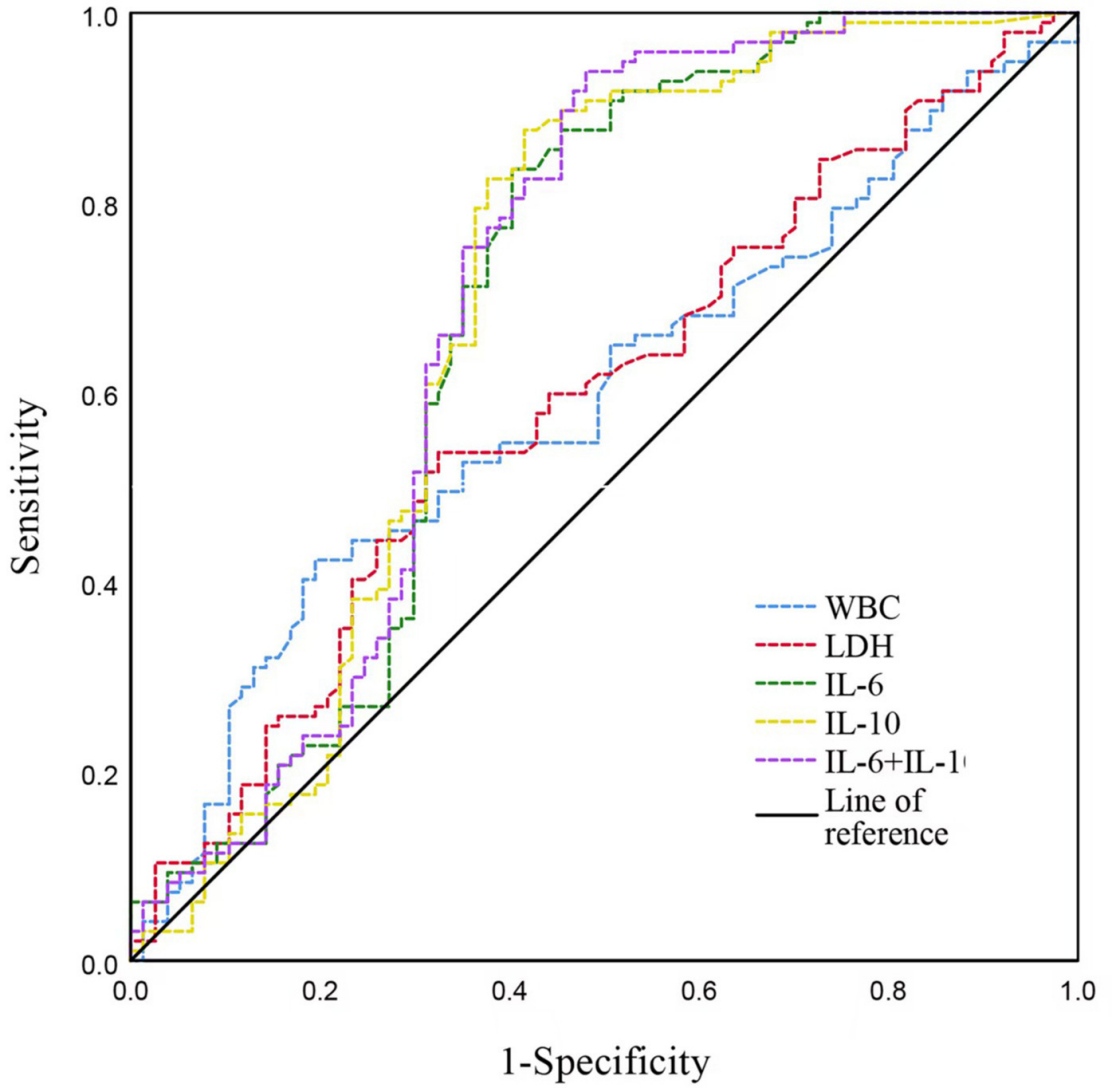
ROC curves illustrating predictive performance of inflammatory biomarkers in children with SMPP. WBC, white blood cell count; ALT, alanine aminotransferase; LDH, lactate dehydrogenase; IL, interleukin.

ROC curve analyses were performed to evaluate the predictive value of inflammatory biomarkers (WBC, LDH, IL-6, IL-10, and the combination of IL-6 and IL-10) for identifying multi-pathogen co-detection in children with SMPP. Both WBC and LDH individually showed moderate predictive performance. IL-6 and IL-10 demonstrated better discriminative capability individually, and combining IL-6 and IL-10 further enhanced diagnostic sensitivity, achieving the highest overall diagnostic accuracy (Table 7, Figure 3).

**Table 7:** ROC curve analysis of inflammatory biomarkers for predicting multi-pathogen co-detection in children with SMPP.

Variable	Cut-off	AUC	95% CI	*P*-value	Sensitivity (%)	Specificity (%)
WBC (×10^9^/L)	8.28	0.60	0.51–0.68	0.023	42.3	80.5
LDH (U/L)	337.5	0.59	0.50–0.67	0.041	53.6	67.5
IL-6 (pg/ml)	37.89	0.69	0.60–0.77	<0.001	83.5	59.7
IL-10 (pg/ml)	14.16	0.69	0.61–0.78	<0.001	87.6	58.4
IL-6 + IL-10	—	0.71	0.62–0.79	<0.001	93.8	52.0

ROC, receiver operating characteristic; AUC, area under the curve; CI, confidence interval.

## 4 Discussion

Previous studies have demonstrated that co-infections with viral and bacterial pathogens frequently occur in pediatric MPP, with reported co-infection rates generally ranging from 30% to 60% (5, 6, 10). Some studies have reported even higher rates, exceeding 88% (11). Clinically, pediatric MPP cases complicated by co-infections typically present with more severe and diverse symptoms, resulting in prolonged hospital stays (6, 10, 12). These findings highlight that co-infection with multiple respiratory pathogens significantly contributes to the severity and clinical complexity of MPP.

In the current prospective observational multicenter study, additional pathogens were detected in 132 of 266 children (49.62%) with MPP, a rate consistent with prior reports (5, 6, 10). Furthermore, the multi-pathogen co-detection rate was significantly higher in the severe group than in the mild group (54.05% vs. 39.51%, *χ*^2^ = 4.769; *P* = 0.033), suggesting that co-infections may be associated with increased disease severity in pediatric MPP.

Interestingly, subgroup analysis by region revealed no significant difference in multi-pathogen co-detection rates between severe and mild cases within each center (Tianjin, Shanghai, Wenzhou, Chengdu; all *P* > 0.05). This is likely attributable to limited statistical power due to smaller sample sizes at each center, combined with the relatively short duration of the study (one winter month). Additionally, similar respiratory pathogens may have concurrently circulated across these regions during the study period, potentially obscuring regional variations. Future larger-scale studies conducted across multiple seasons are warranted to clarify potential geographic differences in co-infection patterns.

Retrospective analyses of 396 children with MPP identified *S. pneumoniae*, *H. influenzae*, and *S. aureus* as the most common bacterial co-infections, while viral co-infections commonly involved human bocavirus (HBoV), RhV, and respiratory syncytial virus (RSV) (6). In the current study, RhV (9.4%), AdV (7.1%), and IAV (6.8%) were the predominant viral pathogens co-detected with MP, *while H. influenzae* (9.8%) and *S. pneumoniae* (6.0%) were the primary bacterial co-pathogens. Some studies identified *S. pneumoniae* as the most common bacterial co-infection (7); however, our study found *H. influenzae* to be predominant, possibly due to seasonal differences and limited sample size.

Indeed, regional variations were notable, with *H. influenzae* (13.33%) being the most common co-pathogen in Tianjin, AdV and IAV (12.65%) dominating in Shanghai, and RhV being prominent in Wenzhou (18.42%) and Chengdu (12.36%). Such differences underscore the importance of considering local epidemiology, environmental factors, and healthcare practices when developing diagnostic and therapeutic strategies.

Given the high frequency and complexity of pathogen co-infections in severe pediatric MPP, there is an urgent need for rapid and cost-effective multiplex diagnostic tools. Recent evidence highlighting neurological dysfunction associated with atypical pathogens such as ammonia-producing microorganisms further underscores the importance of comprehensive pathogen screening in clinical practice (13). Metagenomic next-generation sequencing (mNGS) is of high value in detecting novel, rare, and atypical pathogens (14), but its use in routine practice is limited by its high cost, the influence of human genomic material, and the inability to simultaneously detect both DNA and RNA pathogens in one run (15). Targeted NGS (tNGS), in contrast, uses panels of known pathogen sequences to screen clinical isolates. These panels can target multiple types of pathogens (bacteria, viruses, fungi, atypical agents) with high specificity and sensitivity, and provide faster turnaround times while sequencing directly from clinical specimens (16, 17). In our study, the tNGS panel covered 198 pathogens (over 95% of common respiratory pathogens) and cost roughly one-quarter that of mNGS, making it a practical approach for early and comprehensive pathogen detection in MPP.

MP infection impairs cellular and humoral immunity, leading to an immunosuppressed state that facilitates secondary infections (18). Previous studies have reported elevated levels of inflammatory cytokines, such as IL-1β, IL-6, IL-8, IL-10, and TNF-α, in patients with MPP compared to healthy controls (19, 20). Consistent with these findings, our study showed significantly higher levels of IL-6, IL-10, and IFN-γ levels in severe cases compared to mild cases, with further elevations of IL-6 and IL-10 observed in patients with multi-pathogen co-infections. These results suggest that co-infections intensify inflammatory responses, thereby exacerbating disease severity.

IL-6 and IL-10 play key roles in inflammation and immune regulation, and their marked elevation may reflect more severe disease progression and greater tissue damage. Our further ROC analysis confirmed that IL-6 and IL-10, individually and especially in combination, exhibit high sensitivity (>80%, and approximately 94% combined) for detecting co-infections. Thus, combined IL-6 and IL-10 measurement might be clinically valuable for early identification of SMPP cases at risk of co-infection, facilitating timely intervention and improved patient outcomes.

In addition to cytokines demonstrating significant differences (e.g., IL-6, IL-10, and IFN-γ), several other cytokines, including IL-4, IL-8, and TNF-α, did not differ significantly between the mild and severe groups. This might indicate that these cytokines play a less direct role in the immunopathology of SMPP, or it may reflect sampling timing that did not align with their peak concentrations. Future studies examining the dynamic changes of these cytokines during the course of illness may clarify their roles in MPP progression.

Markers of infection and inflammation such as CRP, ESR, and LDH were also higher in our severe cases than in mild cases, in line with previous findings (18). MP infection activates exogenous and endogenous coagulation systems, leading to coagulation abnormalities and promoting thrombosis (21). Higher D-dimer levels existed in children with SMPP children compared to mild cases in the current study. This indicates a heightened state of coagulation, suggesting that potential thrombotic complications may contribute to increased disease severity and should be carefully considered in the management of SMPP.

In contrast to previous single-center studies or those limited to detecting specific pathogens, our multicenter approach utilizing tNGS provided comprehensive, accurate, and generalizable identification of respiratory co-pathogens in pediatric MPP. Furthermore, our ROC curve analyses of inflammatory biomarkers—particularly the combined use of IL-6 and IL-10—provided novel and valuable diagnostic insights, significantly improving the predictive accuracy for detecting severe cases complicated by co-infections. The integration of clinical characteristics and biomarker analysis further enhances diagnostic precision and holds potential for improving clinical decision-making and patient outcomes.

Several limitations should be noted. The relatively short duration of our study (one winter month) and moderate sample size (*n* = 266) may restrict the generalizability of pathogen prevalence across different seasons and geographic regions. Additionally, distinguishing colonization from infection remains challenging with current tNGS technology, particularly for pharyngeal swab samples, as uniform diagnostic standards are lacking. Although we mitigated this concern by employing BALF samples for severe cases and independent clinical assessments by experienced clinicians, differences in sampling methods remain a limitation. Specifically, pathogen concentrations likely varied between mild (pharyngeal swabs) and severe cases (BALF samples), potentially resulting in higher observed co-detection rates among severe patients. Furthermore, logistic regression analysis was not performed, thus limiting our ability to identify co-detected pathogens as independent predictors of SMPP.

Future studies with larger cohorts, extended durations, and spanning multiple seasons are essential for validating and generalizing our preliminary results. Uniform sampling methods or the concurrent collection of paired upper and lower respiratory tract samples should be considered to accurately explore pathogen co-detection rates and their relationship with disease severity. Further studies employing multivariate logistic regression analysis would clarify whether multi-pathogen co-detection independently predicts SMPP.

In conclusion, this prospective observational multicenter study demonstrated a high prevalence of multi-pathogen co-infections among pediatric patients with MPP, particularly in severe cases. The presence of these co-infections was associated with significantly elevated inflammatory markers, including WBC count, LDH, IL-6, and IL-10, indicating more intense inflammatory responses. Understanding the complex interactions between MP and co-detected pathogens, along with regional and seasonal variations, is critical for improving early diagnosis and targeted management strategies, ultimately enhancing clinical outcomes in pediatric MPP.

## Data Availability

The raw data supporting the conclusions of this article will be made available by the authors, without undue reservation.
